# New congenital anomalies of the kidney and urinary tract and outcomes in *Robo2* mutant mice with the inserted *piggyBac* transposon

**DOI:** 10.1186/s12882-016-0308-5

**Published:** 2016-07-26

**Authors:** Jialu Liu, Li Sun, Qian Shen, Xiaohui Wu, Hong Xu

**Affiliations:** 1 Department of Nephrology and Rheumatism, Children’s Hospital of Fudan University, 399 WanYuan Road, Shanghai, 201102 China; 2 State Key Laboratory of Genetic Engineering and National Center for International Research of Development and Disease, Institute of Developmental Biology and Molecular Medicine, Collaborative Innovation Center for Genetics and Development, School of Life Sciences, Fudan University, Shanghai, 200433 China

**Keywords:** CAKUT, VUR, *Robo2*, *PiggyBac*

## Abstract

**Background:**

Disruption of ROBO2 in humans causes vesicoureteral reflux (VUR)/congenital anomalies of the kidney and urinary tract (CAKUT). *PiggyBac* (PB) is a DNA transposon, and its insertion often reduces—but does not eliminate—gene expression. The *Robo2* insertion mutant exhibited non-dilating VUR, ureteropelvic junction obstruction (UPJO) not found in reported models. We studied the incidence and outcomes of VUR/CAKUT in this mutant and explored the relationship between *Robo2* gene expression and the occurrence and severity of VUR/CAKUT.

**Methods:**

The urinary systems of newborn mutants were evaluated via Vevo 770 micro-ultrasound. Some of the normal animals—and all of the abnormal animals—were followed to adulthood and tested for VUR. Urinary obstruction experiments were performed on mice with hydronephrosis. The histology of the kidney and ureter was examined by light microscopy and transmission electron microscopy. *Robo2*^*PB*/*PB*^ mice were crossed with *Hoxb7*/*myr*-*Venus* mice to visualize the location of the ureters relative to the bladder.

**Results:**

In *Robo2*^*PB*/*PB*^ mice, PB insertion led to an approximately 50 % decrease in Robo2 gene expression. The most common (27.07 %, 62/229) abnormality was non-dilating VUR, and no statistically significant differences were found between age groups. Approximately 6.97 % displayed ultrasound-detectable CAKUT, and these mice survived to adulthood without improvement. No severe CAKUT were found in *Robo2*^*PB*/+^ mice. The refluxing ureters showed disorganized smooth muscle fibers, reduced muscle cell populations, intercellular edema and intracytoplasmic vacuoles in smooth muscle cells. Both UPJ and UVJ muscle defects were noted in *Robo2*^*PB*/*PB*^ mice.

**Conclusions:**

*Robo2*^*PB*/*PB*^ mice is the first *Robo2*-deficient mouse model to survive to adulthood while displaying non-dilating VUR, UPJO, and multiple ureters with blind endings. The genetic background of these mutants may influence the penetrance and severity of the CAKUT phenotypes. VUR and other CAKUT found in this mutant had little chance of spontaneous resolution, and this requires careful follow-up. We reported for the first time that the non-dilated refluxing ureters showed disorganized smooth muscle fibers and altered smooth muscle cell structure, more accurately mimicking the characteristics of human cases. Future studies are required to test the role of *Robo2* in the ureteric smooth muscle.

## Background

Congenital anomalies of the kidney and urinary tract (CAKUT) are the most common causes of renal failure in childhood [1] and represent approximately 30 % of all prenatally diagnosed malformations [2]. CAKUT are phenotypically variable and can affect the kidney(s) and/or the lower urinary tract. Vesicoureteral reflux (VUR) is the most common congenital urinary tract abnormality in childhood and has been associated with an increased risk of urinary tract infection (UTI) and renal scarring, known as reflux nephropathy (RN) [3]. Ureteropelvic junction obstruction (UPJO) is the leading cause of hydronephrosis in children.

CAKUT results from faulty urinary tract development, a process for which numerous genes are critical. ROBO2, a member of the immunoglobulin gene superfamily, is a receptor for the ligand SLIT2. SLIT2-ROBO2 signaling is important for axon guidance during development [4], and both human studies and animal models have provided evidence for an association between the interruption of ROBO2 and VUR/CAKUT [5–9].

Lu et al. report the disruption of *ROBO2* in one patient with a balanced translocation and a phenotype that included VUR, ureterovesical junction (UVJ) defects, and megaureter [6]. In addition, heterozygous point mutations in *ROBO2* have been identified in many patients with nonsyndromic VUR [6, 10]. However, some studies [11–14] have been unable to validate the association between *ROBO2* and human VUR. Heterozygous mutations have recently been identified in patients with UPJO, posterior urethral valve (PUV) or multicystic dysplastic kidney (MCDK) [15].

*Robo2*-deficient mouse models have been used to explore the pathogenesis of *Robo2* in CAKUT [5–7]. Studies have shown that SLIT2-ROBO2 signaling plays a key role both in normal ureteric budding and in the reciprocal induction of nephrogenesis by negatively regulating GDNF/RET activity [5]. *Robo2* homozygotes had multiplex, dysplastic kidneys and short ureters and died within 48 h of birth [5–7]. Possibly due to variable *Robo2* gene dosage, the mosaic mice had broad-spectrum CAKUT phenotypes including renal dysplasia, short ureter, megaureter, abnormally located UVJ and hydronephrosis [6, 7]. Of the long-surviving heterozygotes, 4/26 (15 %) exhibited unilateral megaureter and a wide-open UVJ [6]. Other phenotypes reported in patients with *ROBO2* mutations, such as UPJO, PUV and non-dilating VUR, were not found in these models.

*PiggyBac* (PB) is a DNA transposon. Its insertion often reduces but does not eliminate gene expression, facilitating the identification of specific genetic functions [16], and it is a mutagen suitable for large-scale mutagenesis in mice. The Institute of Developmental Biology and Molecular Medicine at Fudan University performed a large-scale PB insertional mutagenesis and identified one *Robo2* insertion mutant. This mutant was both viable and fertile, and it exhibited non-dilating VUR, UPJO, and ureterovesical junction obstruction (UVJO) that were not found in reported models or other CAKUT phenotypes. We study the incidence and outcomes of VUR/CAKUT in this mutant and explore the relationship between *Robo2* gene expression and the occurrence and severity of VUR/CAKUT.

## Methods

### Mouse procedure

The PB transposon was inserted in the first intron of *Robo2* on mouse chromosome 16, nucleotide 74379378. The insertion direction was opposite the gene location. *Robo2*^*PB*/*PB*^ mice were crossed with *Hoxb7*/*myr*-*Venus* mice, which have been described previously [17], to visualize the location of the ureters relative to the bladder, as performed previously [18, 19].

### Mapping the PB-inserted *Robo2* allele

Offspring with the transposon inserted into the *Robo2* gene were genotyped by polymerase chain reaction (PCR) amplification using the primers P3 (5′-CTGAGATGTCCTAAATGCACAGCG-3′), P4 (5′-AAGATTCCCTTTCCATGCAGAAGAG-3′) and P5 (5′-GTGTACAGGAGTTTGGCACTGG-3′). *Robo2*^*PB*/*PB*^ and *Robo2*^*PB*/+^ were amplified by P3 and P5, which produced a 614 bp fragment. Wild type and *Robo2*^*PB*/+^ were amplified by P4 and P5, which yielded an 864 bp fragment. PCR conditions were as follows: initial denaturation at 93 °C for 90 s; 40 cycles of 93 °C for 30 s, 57 °C for 30 s, and 65 °C for 2 min; and a final extension at 65 °C for 10 min. Genomic DNA extracted from mouse toes was used as template.

### Ultrasound investigation of the urinary system

The urinary system was examined by a Vevo 770 high-frequency micro-ultrasound system. Mice were anesthetized with 2.5 % isoflurane in 1 L/min oxygen via nose cone. A heat lamp was used to keep mice warm during anesthesia. A chemical depilatory was used to remove the fur above the position of the kidneys, and ultrasound gel was generously applied directly to the depilated surface. Next, the real-time micro visualization (RMV) transducer was placed on the ultrasound gel along the spine to identify the kidneys. RMV-708 was used for newborn mice, and RMV-712 was used for juvenile and adult mice. Grossly observable phenotype were described as severe CAKUT.

### VUR and urinary tract obstruction experiments

Operators who were blinded to the genotypes performed the VUR test. Mice were dissected using an anterior midline incision to expose the kidneys and the urinary tract. The bladder was punctured with a 25 gauge needle to manually inject methylene blue (1 mg/ml in PBS) at a rate of 100 μl/min until the dye exited through the urethra [20]. The severity of VUR was determined by the extent of the ureter dilation. Combined with ultrasound results, VUR was divided into dilating VUR and non-dilating VUR. To test for urinary tract obstruction, the renal pelvis was microinjected with dye, and its passage along the urinary tract was monitored to determine whether there was evidence of impaired flow along the tract [20].

### Histopathological analysis

Kidneys with attached ureters and bladders were removed from the euthanized mice. Tissue samples were fixed in 4 % paraformaldehyde, embedded in paraffin, sectioned at 4 μm, stained with hematoxylin and eosin (H & E) and Masson’s trichrome and examined under a light microscope.

Sections of the kidney from wild type and *Robo2*^*PB*/*PB*^ mice (VUR and renal dysplasia) were immunostained for ROBO2 (the EnVision method). ROBO2 was detected using a rabbit polyclonal anti-ROBO2 antibody (1:50; Santa Cruz Biotechnology, Inc.).

For transmission electron microscopy, ureters and renal cortices were cut into small pieces (<1 mm^3^) and rapidly fixed in 2.5 % glutaraldehyde, then washed in phosphate buffer (pH 7.4), post-fixed in 1 % osmium tetroxide in the same phosphate buffer and dehydrated in increasing concentrations of alcohol and embedded in araldite. The ultrathin 70 nm sections were cut on an ultramicrotome, collected on copper grids, stained with uranyl acetate and lead citrate, and examined by transmission electron microscopy (Philips CM120). RFP and GFP fluorescence were monitored and photographed using an epifluorescence stereomicroscope (MZFLIII; Leica).

### Real-time quantitative PCR (qRT-PCR)

The human metanephric kidney is functional at approximately 11 gestational weeks, which corresponds to embryonic stages E15.5-E16.5 in mice. Total RNA from E15.5 embryos was extracted using TRIzol (Invitrogen, USA), and cDNA was prepared with the RNA PCR Kit (TaKaRa). The mixture was incubated for 15 min at 37 °C and 5 s at 85 °C. The qRT-PCR was performed with an All-in-One qPCR Mix Detection Kit (GeneCopoeia) on the Mx3000P Quantitative PCR System (Stratagene) to detect *Robo2* expression with the primers P1/P2 located within exons 1 and 2. GAPDH was used as an internal control. The reaction conditions were set as follows: an initial denaturation step of 10 min at 95 °C followed by 40 cycles (10 s at 95 °C, 20 s at 60 °C, and 15 s at 72 °C) with a final elongation step of 5 min at 72 °C. Quantification was performed by measuring the threshold cycle (CT value) and using a standard curve as a reference. The primer sequences were as follows: 5′-GCGGATCTTTATTCTTTTTGCG-3′ (sense) and 5′-TCCTTTTTCCAGTAGATGGTTG-3′ (antisense) for *Robo2*, and 5′-TGTTCCTACCCCCAATGTGTCC-3′ (sense) and 5′-GGAGTTGCTGTTGAAGTCGCAG-3′ (antisense) for GAPDH.

### ROBO2 protein analysis

Kidney and brain proteins were extracted in RIPA buffer with freshly added 1 mM PMSF and 1× proteinase inhibitor (Roche). Next, they were quantified with the BCATM Protein Assay Kit (Pierce). Equal protein were separated by SDS/PAGE and transferred onto PVDF membranes. ROBO2 expression was detected using an anti-ROBO2 antibody (ab64158; 1:1,000) as the primary antibody and biotin-conjugated goat anti-rabbit IgG (CoWin Biotech Co; 1:10,000) as the secondary antibody. SuperSignal West Pico Chemiluminescent Substrate (Thermo 34080) was used for detection and GAPDH levels were used as loading controls (CoWin Biotech Co; 1:500).

### Statistical analysis

All of the statistical analyses were performed using SPSS Version 19. A statistical analysis of the RNA results was performed using Student’s *t*-test. The prevalence of categorical variables was analyzed using the Chi-square test. Statistical significance was set at *P* < 0.05.

## Results

### Genetic characterization of *Robo2* mutant mice

**Fig. 1:**
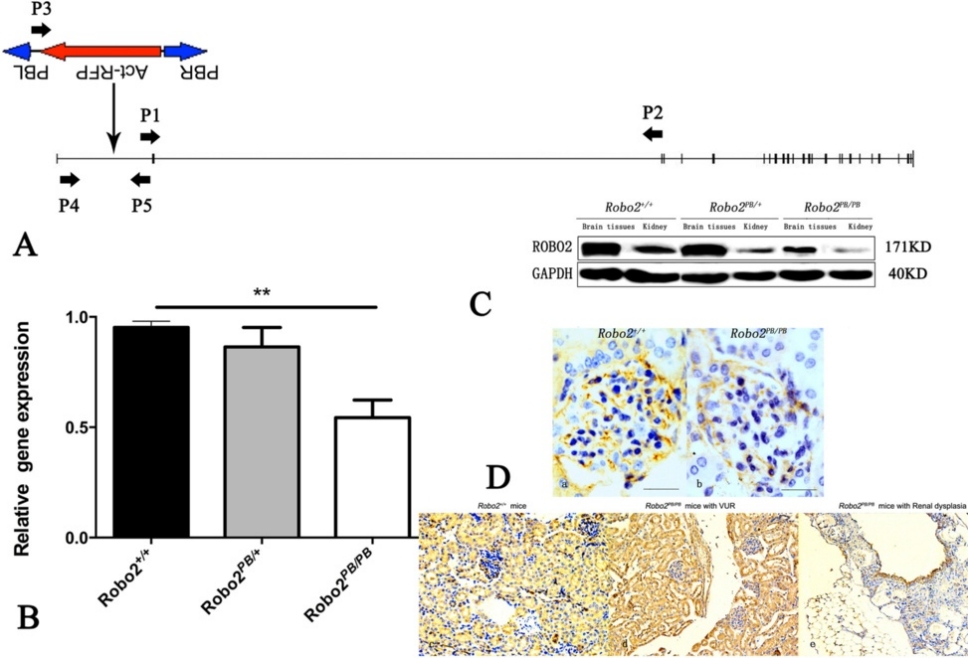
Characterization of the PB insertion in *Robo2* mutant mice. **a** Position of the PB insertion. The PB integration site in *Robo2* ^*PB*/*PB*^ mice mapped to the first intron of *Robo2* on mouse chromosome 16, nucleotide 74379378. Diagram shows the genomic structure of the *Robo2* gene with exons (vertical lines) and introns (transverse lines). Gene-specific primers are indicated. **b** Disruption of *Robo2* transcript in *Robo2* mutants. *Robo2* transcripts were analyzed by quantitative PCR using the indicated primers P1/P2. The cDNAs from whole embryos at E15.5 were amplified as templates. GAPDH was used as an internal control. **P < 0.01, *n* = 3 per group. **c** Western blots were performed on the brain and kidneys from wild-type and *Robo2* ^*PB*/*PB*^ mice. The ROBO2 expression level decreased in *Robo2* ^*PB*/*PB*^ mice. **d** Expression of ROBO2 in the kidneys. a and c :expression of ROBO2 in the kidneys from *Robo2* ^+/+^ mice; b: expression of ROBO2 in the kidneys from *Robo2* ^*PB*/*PB*^ mice; d: expression of ROBO2 in the kidneys from *Robo2* ^*PB*/*PB*^ mice with VUR; e: expression of ROBO2 in the kidneys from *Robo2* ^*PB*/*PB*^ mice with renal dysplasia

The PB transposon was inserted into the first intron of the *Robo2* gene (Fig. 1a). In E15.5 embryos, quantitative PCR showed that transcript levels from homozygotes (*Robo2*^*PB*/*PB*^) were reduced by approximately 50 % compared to wild-type (*Robo2*^+/+^) littermates. There was no significant difference between heterozygotes (*Robo2*^*PB*/+^) and wild-type littermates (Fig. 1b). Western blots showed that ROBO2 protein levels were decreased in the brain tissues and kidneys of adult *Robo2*^*PB*/*PB*^ mice (Fig. 1c), consistent with the quantitative PCR results. Expression of ROBO2 in the control kidneys was higher than in *Robo2*^*PB*/*PB*^ mice. Expression of ROBO2 in the refluxing kidney was higher than in the kidney of renal dysplaisa (Fig. 1d).

### The incidence of urinary malformation in *Robo2* mutant mice

The urinary systems of all the newborn *Robo2* mutant mice were evaluated using a Vevo 770 micro-ultrasound. Some of the normal mice and all of the abnormal mice were followed to adulthood. All mice were tested for VUR. Urinary obstruction experiments were performed on the mice with hydronephrosis after the VUR test.”

**Fig. 2:**
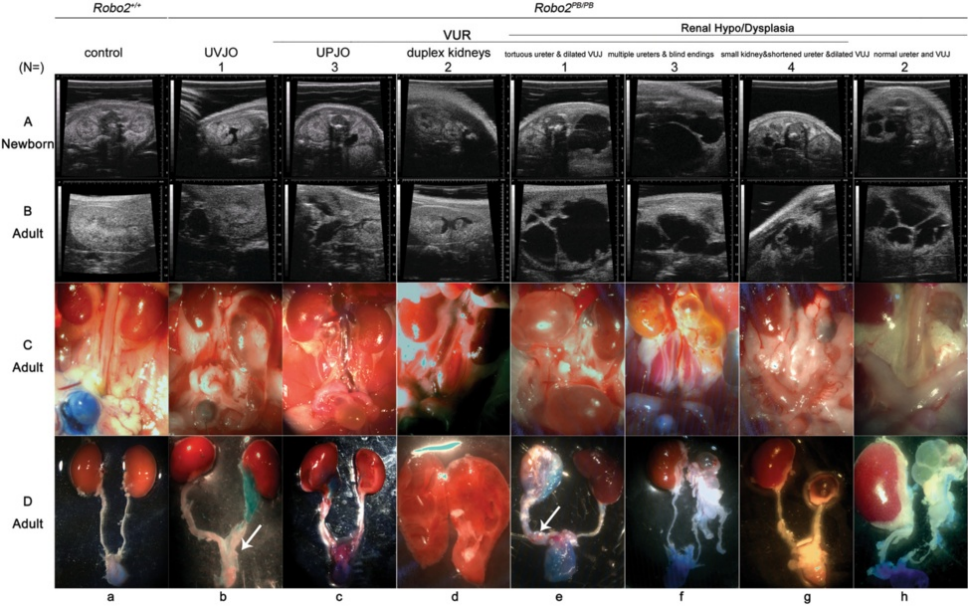
Ultrasound images and gross anatomy of urinary malformations found in *Robo2* ^*PB*/*PB*^ mice. **a** Ultrasound images depict the urinary tract system in wild-type (*a*) and *Robo2* ^*PB*/*PB*^ newborn mice (*b*–*h*). **b** Ultrasound detection of the urinary tract system in wild-type (*a*) and *Robo2* ^*PB*/*PB*^ adult mice (*b*–*h*). **c** Macroscopic morphology of the urinary tract system in adult mice. *Robo2* ^*PB*/*PB*^ mutant mice develop diverse urinary malformations (*b*–*h*). **d** Gross structure of the urinary tract system from adult mice. Arrows indicate abnormal UVJs, and arrowheads indicate abnormal ureters

**Fig. 3:**
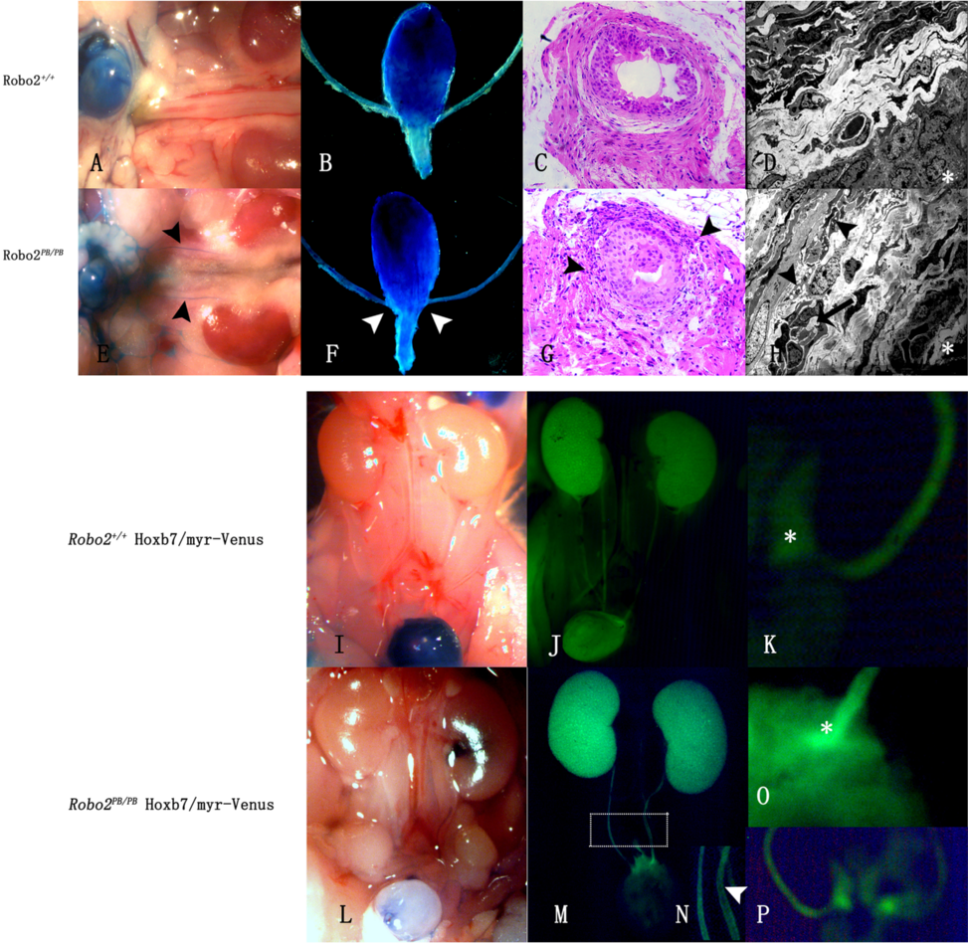
VUR experiment and histopathological analysis of the UVJ from *Robo2* ^*PB*/*PB*^ and wild-type mice. **a**–**d** VUR experiment and histopathological analysis of intravesical ureters in a control mouse. **e** Bilateral VUR was observed in a *Robo2* ^*PB*/*PB*^ mouse. **f** Macroscopic morphology of ureters and bladders was normal. The UVJs (*arrowheads*) were similar to the wild-type control (**b**). **g** Disorganized smooth muscle fibers (*arrowheads*) were found in intravesical ureter sections. **h** Electron microscopy of the intravesical ureters from *Robo2* ^*PB*/*PB*^ mice with VUR showed normal overall structure but decreased muscle cell population, along with intercellular edema in the muscular layer (*arrow*) and intracytoplasmic vacuoles in smooth muscle cells (*arrowheads*). **i**–**k** VUR experiment and histopathological analysis of intravesical ureters were analyzed in a *Robo2* ^+/+^; *Hoxb7*/*myr*-*Venus* control mouse. **l** Unilateral VUR was observed in a *Robo2* ^*PB*/*PB*^; *Hoxb7*/*myr*-*Venus* mouse. **m** The ureter was mildly dilated on the left in a *Robo2* ^*PB*/*PB*^; *Hoxb7*/*myr*-*Venus* mouse with left VUR. **n** Higher magnification of the boxed region in M, demonstrating a wilder inner diameter in the refluxing ureter (*arrow head*). **o**-**p** The connection of the refluxing ureter relative to the bladder appeared normal in a *Robo2* ^*PB*/*PB*^; *Hoxb7*/*myr*-*Venus* mouse with VUR. Magnifications: (**c** and **g**) 400; (**d** and **h**) 1,500. *Asterisks* in **d** and **h** indicate the mucosa of the ureter

The Vevo 770 micro-ultrasound showed that 6.97 % (16/229) of newborn *Robo2*^*PB*/*PB*^ mice (8 males, 8 females) displayed diverse urinary malformations, including hydronephrosis, duplex kidneys and dilated ureter (Panel A from Fig. 2). They survived to adulthood without improvement (Panel B from Fig. 2). Compared to wild-type mice (Fig. 3a–d, i–k), non-dilating VUR was more requent in *Robo2*^*PB*/*PB*^ mice (Fig. 3e–h, l–p) (27.07 % versus 6.84 %, *P* = 0.000, Chi-squared = 19.65). There were no differences in simple VUR prevalence between age < 60 days and age > 60 days (27.07 % versus 32.50 %, *P* > 0.05, Chi-squared = 0.71) in *Robo2*^*PB*/*PB*^ mice, and no gender (*P* > 0.05, Chi-squared = 0.42) or side (*P* > 0.05, Chi-squared = 0.70) biases were observed. In summary, urinary malformations were found in 34.06 % (78/229) of *Robo2*^*PB*/*PB*^ mice, and non-dilating VUR (27.07 %, 62/229) was recognized as the most common abnormality. Grossly identifiable urinary malformations were not found in heterozygotes (*N* = 127).

### Gross anatomy, histology and outcomes of the affected ureters and kidneys in *Robo2*^*PB*/*PB*^ mice with non-dilating VUR

Compared to the histopathological analysis of the intravesical ureter in a wild-type mouse without VUR (Fig. 3b–d), normal UVJ location and ureteral orifice without dilation (Fig. 3f) were observed in a *Robo2*^*PB*/*PB*^ mouse with bilateral VUR. Paraffin-embedded sections of refluxing ureters showed normal gross structure, but contained disorganized smooth muscle fibers at the level of the bladder (arrowheads in Fig. 3g). In addition, urothelium was found to partially occlude the lumen, but cellular atypia was not obvious, and cell layer was similar to that of the control, so malignant hyperplasia was not considered (Fig. 3g). Examination by transmission electron microscopy showed both normal general structure and reduced muscle cell population (Fig. 3h). The muscle layer showed intercellular edema (arrow in Fig. 3h) and intracytoplasmic vacuoles (arrowheads in Fig. 3h) in smooth muscle cells.

For a more detailed analysis of the kidney and ureter abnormalities, kidneys and ureters from newborn *Robo2*^+/+^; *Hoxb7*/*myr*-*Venus* (Fig. 3i-k) and *Robo2*^*PB*/*PB*^; *Hoxb7*/*myr*-*Venus* (Fig. 3l-p) mice were visualized by fluorescence microscopy. The kidneys were normal in size, and inner diameter of the refluxing ureter from a *Robo2*^*PB*/*PB*^; *Hoxb7*/*myr*-*Venus* mouse was wilder than the contralateral ureter (Fig. 3m-n). However, the connection of the ureter relative to the bladder appeared normal (Fig. 3o–p).

**Fig. 4:**
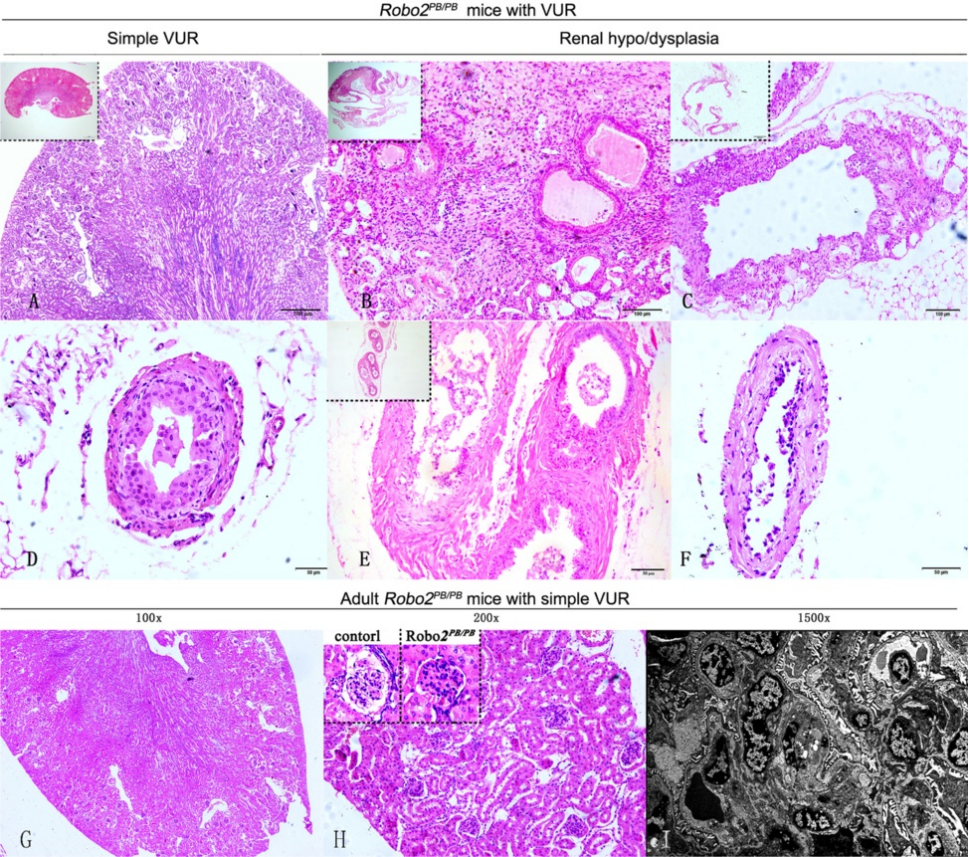
Histopathological analysis of kidneys and ureters from *Robo2* ^*PB*/*PB*^ mice with VUR. **a** Masson staining of a kidney from a newborn *Robo2* ^*PB*/*PB*^ mouse with simple VUR revealed no scarring. **b** Kidney section from a *Robo2* ^*PB*/*PB*^ mouse with hydronephrosis accompanied by multiple ureters depicted dilatation of the collecting tubule, thyroidization-type tubular atrophyimmature glomeruli, and abnormal capillaries surrounded by loose, undifferentiated mesenchymal tissue (**c**) Kidney section from a *Robo2* ^*PB*/*PB*^ mouse with an abnormally small kidney accompanied by short dilated ureter depicted nearly complete loss of renal parenchyma, cyst formation and focal glomerular capillary tuft collapse (**d**) H&E staining of a ureter from a *Robo2* ^*PB*/*PB*^ mouse with simple VUR revealed normal overall structure. **e** Multiple ureters from the same mouse as in B showed a thin layer of surrounding mesenchyme. **f** Ureter from the same mouse as in C showed a thin layer of ureter epithelium. **g** H&E staining of kidneys from adult *Robo2* ^*PB*/*PB*^ mice with bilateral VUR. **h** H&E staining of kidneys showed glomerular hypertrophy and no inflammation. **i** Electron microscopy showed normal glomerular structure. Scale bars: 5,00 μm in A; 1,00 μm in **b**–**c**; 50 μm in **d**–**f**

To assess the progression of renal damage, we evaluated kidneys from adult *Robo2*^*PB*/*PB*^ mice with VUR and found no signs of RN (Fig. 4g–h). The kidneys were examined by transmission-electron microscopy, revealing a normal glomerular structure (Fig. 4i).

### Gross anatomy, histology and outcomes of the affected ureters in *Robo2*^*PB*/*PB*^ mice with other CAKUT phenotypes

Dissection revealed that *Robo2*^*PB*/*PB*^ mice had diverse CAKUT phenotypes (Table 1), including UVJO (Fig. 2b), UPJO (Fig. 2c), duplex kidneys (Fig. 2d) and renal hypo/dysplasia (Fig. 2e-h). No obvious malformations of the genital tracts were observed in any genotype, and different phenotypes were observed within one litter. Of the grossly abnormal phenotypes, 62.5 % (10/16) were on the left, 37.5 % (6/16) were on the right, and none were bilateral.

**Table 1:** Penetrance of CAKUT in Robo2 mutant mice

	CAKUT penetrance (%)	VUR		Renal Hypo/dysplasia	Hydronephrosis	DS/multiple e ureter	UPJO	UVJO
		Dilating VUR	Simple VUR					
*Robo2* ^*del5*/*del5*^	20/20 (100 %)-14/14 (100 %)	Not reported	Not reported	20/20 (100 %)	Not reported	12/14 (86 %)	Not reported	Not reported
*Robo2* ^*del5*/+^	4/26 (15 %) Unilateral CAKUT-VUR	4/26 (15 %) megaureter and a wide- open UVJ,	Not reported	Not reported	Not reported	Not reported	Not reported	Wide-open UVJ and megaureter
*Robo2* ^*del5*/*del5*^↔*Robo2* ^*del5*/*flox*^ *mosaic*	4/10 (40 %)-7/10 (70 %) unilateral CAKUT	Not reported	Not reported	Large cyst	Severe hydronephrosis and short ureter megaureter	Not reported	Not reported	Bilateral UVJ defects, located laterally and cephalad in the bladder
*Robo2* ^*PB*/*PB*^	78/229 (34.06 %)	13/229 (5.68 %)	62/229 (27.07 %,)	10/229 (4.37 %)	8/229 (3.49 %)	5/229 (2.18 %,)	3/229 (1.31 %)	1/229 (0.44 %)
wild-type (*Robo2* ^+/+^)	8/117 (6.84 %)	0	8/117 (6.84 %)	0	0	0	0	0

Because there were only a small number of such mice, the results obtained with newborn *Robo2* ^+/+^; *Hoxb7*/*myr*-*Venus* and *Robo2* ^*PB*/*PB*^; *Hoxb7*/*myr*-*Venus* mice were not included

*DS* duplex collecting system, *UPJO* ureteropelvic junction obstruction, *UVJ* ureterovesical junction obstruction, *VUR* vesicoureteral reflux

The anatomy of the renal hypo/dysplasia in *Robo2*^*PB*/*PB*^ mice was characterized by two distinct patterns. One was hydronephrosis with a markedly dilated renal calyx and nearly complete loss of renal parenchyma (Fig. 2e–f and h). The other was an abnormally small kidney (Fig. 2g). Renal hypo/dysplasia was likely to coexist with other phenotypes (Fig. 2e–h). The dysplastic kidney was grossly cystic, and the contralateral kidney appeared grossly normal with no evidence of hydronephrosis.

Overall, 13/15 *Robo2*^*PB*/*PB*^ mice with grossly identifiable urinary malformations were found to have concomitant VUR. VUR was not observed in one mouse with renal hypo/dysplasia or in one mouse with malformed UVJs located laterally in the bladder (Fig. 2b). The VUR test was unsuccessful in one mouse that had severe malformation of the urinary tract.

**Fig. 5:**
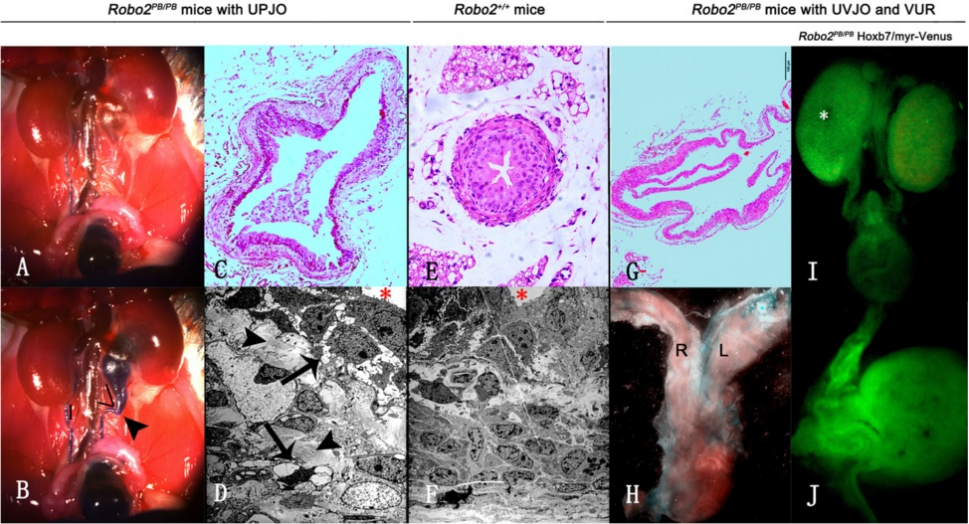
Gross anatomy and histopathological analysis of ureters from *Robo2* ^*PB*/*PB*^ mice with UPJO and UVJO. **a** VUR testing of a hydronephrotic *Robo2* ^*PB*/*PB*^ mouse indicated contralateral VUR. **b** VUR testing then indicated ipsilateral VUR. Dilation of the pelvis is shown. Compared to contralateral normal UPJ (no angle), sharp narrowing (*acute angle*) were found at the UPJ (arrowhead). **c** A proximal ureter section depicted ureter dilation and smooth muscle distortion. **d** Separated muscle cells with intracytoplasmic vacuoles (*arrows*) and excessive collagen (*arrowheads*) were visualized using electron microscopy. **e**–**f** Histopathological analysis of a kidney with ureter from a control mouse. **g** Histological analysis confirmed the dilation of the distal left ureter in the mutant and showed a thin layer of smooth muscle. **h** Both UVJs connected to a caudal site in the bladder close to the urethra, causing dilated ureters. **h** A *Robo2* ^*PB*/*PB*^; *Hoxb7*/*myr*-*Venus* newborn mouse showing a dilated left ureter and dysplastic cyst in the left kidney. Kidneys and ureters were visualized using the *Hoxb7*/*myr*-*Venus* transgene. **i** The left UVJ connected to a caudal site in the bladder close to the urethra, causing a dilated ureter. **j** Higher magnification of abnormal left UVJ in panel **i**. Magnifications: (**c** and **e**) 100; (**d** and **f**) 1,500; (**g**) 200. *Red asterisks* in **d** and **f** indicate the mucosa of the ureter. *White asterisks* in **i** indicates dysplastic cyst

In one mouse with unilateral hydronephrosis, reflux was first found in the contralateral ureter (Fig. 5a), and ipsilateral VUR (Fig. 5b) was then noted to occur with hydronephrosis. The affected mice displayed dilated renal pelvis without any dilatation of the ureter or the UVJ, indicating stenosis at the level of the UPJ. We subsequently examined the histology of the UPJ. The UPJ sections showed dilated ureters and distortion of the smooth muscle (Fig. 5c) compared to UPJ sections of wild-type mice (Fig. 5e). Transmission electron microscopy revealed separated muscle cells and excessive collagen in the UPJ (Fig. 5d).

The UVJ in *Robo2*^*PB*/*PB*^ mice with UVJO connected to a caudal site in the bladder close to the urethra, causing dilated ureter (Fig. 5h). Histological analysis confirmed the dilation of the distal ureter in the mutant and showed a thinner or a thicker layer of smooth muscle (Fig. 5g). Kidneys and ureters were illuminated because of the *PB* [*Act*-*RFP*] transgene and the *Hoxb7*/*myr*-*Venus* transgene. A *Robo2*^*PB*/*PB*^; *Hoxb7*/*myr*-*Venus* newborn mouse exhibited dysplastic cysts in the left kidney, dilated left ureter, and abnormal left UVJ (Fig. 5i-j).

## Discussion

We described a new *Robo2*-deficient mouse model in the FVB/N background. FVB/N is a VUR-resistant strain [21]. A PB insertion partially disrupted the expression of *Robo2*, decreasing it to 50 % of the normal level. The realtime PCR results only showed a downward trend in Robo2 transcription in *Robo2*^*PB*/+^ mice, possibly due to limited data. *Robo2*^*PB*/*PB*^ mice are the first *Robo2*-deficient mouse model to survive to adulthood while displaying non-dilating VUR, UPJO, and multiple ureters with blind endings. Most of these phenotypes have been reported in patients with ROBO2 variants [6, 10, 15, 22]. These urinary malformations mimicked human CAKUT and did not follow a typical Mendelian pattern, as do most CAKUT found in children. More than one CAKUT phenotype can be observed in the same individual (human or mouse), demonstrating the need for a careful assessment of the kidneys and urinary tracts [19]. We performed ultrasound screening and followed abnormal mice to adulthood. Because the prevalence of severe CAKUT phenotypes was relatively low, this approach improved the utility of this animal model. This analysis also indicated that *Robo2*^*PB*/*PB*^ mice preferentially developed unilateral, severe urinary malformations with little chance of spontaneous resolution. Because *Robo2* has also been reported to play roles in the development of the brain [23], lungs [24] and heart [25], we examined the morphology of these organs, but we did not observe any obvious defects (data not shown).

The prevalence of urinary malformations in *Robo2*^*PB*/*PB*^ mice was 34.06 %, higher than the 7.87 % in *Robo2*^*PB*/+^ mice and the 6.84 % in wild-type mice. Non-dilating VUR was the most common abnormality. Approximately 6.97 % of *Robo2*^*PB*/*PB*^ mice displayed severe CAKUT phenotypes that were not found in *Robo2*^*PB*/+^ mice. After dissection, *Robo2*^*PB*/*PB*^ mice showed renal hypo/dysplasia at a rate of 4.37 % renal hypo/dysplasia, duplex systems at 2.18 % duplex system and VUR and other urinary malformations at 31.44 % VUR and other urinary malformations were found in *Robo2*^*PB*/*PB*^ mice. Conventional Robo2 mutants with severe degrees of gene deletion showed severe CAKUT bilaterally and with a higher incidence CAKUT [6]. Eliminating the effect of strain and genetic background differences, our results may suggest that the expression level of Robo2 (type of mutation) influences the penetrance and severity of VUR/CAKUT.

Primary VUR is a congenital defect with an UVJ anomaly. In secondary VUR, the valvular mechanism is intact and healthy to start with but becomes overwhelmed by raised vesicular pressures associated with obstruction. The obstructions may be anatomical or functional. UVJ histological defects were noted in *Robo2*^*PB*/*PB*^ mice. Grossly identifiable urinary malformations with concomitant VUR are unilateral in our models. Therefore, we assume that the VUR found in our models are primary VUR.

Additional factors might predispose mice to diverse urinary malformations. Genetic factors may be influenced by environmental exposure, leading to differing phenotypes despite identical gene mutations [26, 27]. Studies are needed to identify environmental factors that influence the penetrance and severity of CAKUT.

For a more detailed analysis of the kidney and ureter abnormalities, light microscopy evaluation and transmission electron microscopy, respectively, were used. *Robo2*^*PB*/*PB*^ mice were crossed with *Hoxb7*/*myr*-*Venus* mice to visualize the location of the ureters relative to the bladder. The results were compared with the controls and previously published results.

Unlike the dilated and incompetent UVJ that led to high-grade VUR and hydronephrosis in most *Robo2*^*del5*/*del5*^ mice (5), most *Robo2*^*PB*/*PB*^ mice with simple VUR had normal macroscopic UVJ morphology and lacked hydronephrosis. We found disorganized smooth muscle fibers with intercellular edema and decreased numbers of muscle cells with intracytoplasmic vacuoles in the refluxing intravesical ureters. Most severe CAKUT phenotypes were accompanied by VUR. The ureter histological defects were severe in mice with VUR and renal hypo/dysplasia. Reduced and irregular ureteral smooth muscle staining was reported in *Robo2*^*del5*/*del5*^ homozygotes before ureteral dilatation [8]. This mutant is the first mutant in which the histology of the refluxing ureter was similar to the following findings from the intravesical ureteric specimens of VUR patients: smooth muscle disarrangement [28, 29] and intercellular edematous and intracytoplasmic vacuoles [30, 31]. A significant correlation between the grades and smooth muscle lesions [31, 32], widely absent smooth musculature [33], and a replacement of muscle bundles with connective tissue [34] have also been reported in VUR patients. These structural changes may result in the deformation of the architecture of the ureteric wall smooth muscle layer.

Paraffin sections of refluxing *Robo2*^*PB*/*PB*^ mouse kidneys displayed no signs of inflammation or scarring. A previous study demonstrated that sterile reflux alone fails to induce reflux nephropathy [35]. This may explain why our mutants showed no signs of inflammation or scarring. Structural alteration in the podocyte foot process was found in a *Robo2* podocyte-specific knockout mouse [8]. Our electron micrograph shows normal glomerular structure that may be due to the remaining expression of ROBO2.

It has been reported that chronic kidney damage is significantly related to congenital renal hypo/dysplasia, rather than to infections and VUR [36, 37]. Genetic factors may influence both the occurrence of dysplasia and the propensity for scar formation [37]. In our models, 4.37 % of homozygous mutants had histologically demonstrated renal hypo/dysplasia. Some of these may be secondary effects of hydronephrosis; however, some maybe congenital and require careful followed-up.

Both UPJ and UVJ histological defects were noted in *Robo2*^*PB*/*PB*^ mice. Histopathological analysis revealed dilation of the ureter, distortion of the smooth muscle, separated muscle cells with intracytoplasmic vacuoles and excessive collagen. These pathological findings may be secondary effects of hydronephrosis.

The ureteral smooth muscles play a crucial role in ureteral peristalsis [38] and UVJ formation [39]. Sufficient contraction of the ureteric muscular layer by closing the ureteric orifice prevents VUR [40]. It was speculated that the disorganization of the muscular layer leads to a malfunctioning valve system and causes VUR [32]. One study has indicated that congenital obstructions might be due to aberrant ureteric smooth muscle organization, resulting in impaired peristalsis [41]. Future studies are required to provide additional mechanistic insights into the role of *Robo2* in ureteric smooth muscle cells in the development of UPJ and UVJ. Mechanistic studies investigating the role of *Robo2* in smooth muscle cell function, proliferation, and migration are needed. Based on previous studies [42–44], we speculate that ROBO2 in ureteric smooth muscle cells interacts with Ab1, permitting Enabled (Ena)/vasodilator-stimulated phosphoprotein (VASP) protein phosphorylation and thus participating in the cytoskeletal changes in smooth muscle α-actin.

VUR could be caused by defects in ureteric budding, ureter differentiation and elongation, peristalsis, UVJ formation, and bladder and urethra development [45]. Mackie and Stephens proposed the ureteric bud theory [46] that the UB must emerge from the ND at the precise site to invade the MM for normal kidney development to occur. If budding occurs rostral or caudal to this site, would lead to renal hypoplasia/dysplasia and ectopic insertion of the ureter into the bladder, resulting in VUJ obstruction and VUR. Multiple ureteric buds may lead to short duplex ureters, duplex kidneys, ectopic and dysplastic kidneys [45]. We speculate that quantitative differences in either *Gdnf* or genes within the *Robo2*/*Slit* network are most likely responsible for the urinary tract defects we observe. Other factors such as environment factors, may conribute to this process.

There are several limitations to this study. We did not examine how the different anomalies arose, and more work is needed to explain why *Robo2*^*PB*/*PB*^ mice preferentially develop unilateral severe urinary malformations.

## Conclusions

The genetic background of the *Robo2* mutants discussed may influence the penetrance and severity of the CAKUT phenotypes. Genetic factors may be influenced by environmental exposure, leading to differing phenotypes. This mutant is the first *Robo2*-deficient mouse model to survive to adulthood while displaying non-dilating VUR, UPJO, and multiple ureters with blind endings. VUR and other CAKUT in this mutant showed very low rates of spontaneous resolution after birth. We may uncover more deleterious mutations in the *Robo2* gene if we focus on the DNA analysis of patients with these phenotypes. CAKUT associated with genetic mutations may indicate poor prognosis, and patients with *Robo2* mutations should therefore be followed up closely. We first reported that the non-dilated refluxing ureters showed disorganized smooth muscle fibers and altered smooth muscle cell structure, more accurately mimicking the characteristics of human cases. Future studies are required to test the role of *Robo2* in ureteric smooth muscle. *Robo2*^*PB*/*PB*^ mice could be used as an animal model to better understand both urinary tract development and the cellular and molecular mechanisms of *Robo2* in the kidney.

## Abbreviations

CAKUT, Congenital anomalies of the kidney and urinary tract; H & E, hematoxylin and eosin; MCDK, multicystic dysplastic kidney; PCR, polymerase chain reaction; PUV, posterior urethral valve; qRT-PCR, Real-time quantitative PCR; RMV, real-time Micro Visualization; RN, reflux nephropathy; UPJO, Ureteropelvic junction obstruction; UTI, urinary tract infection; UVJ, ureterovesical junction; VUR, Vesicoureteral reflux
